# Machine learning models for assessing risk factors affecting health care costs: 12-month exercise-based cardiac rehabilitation

**DOI:** 10.3389/fpubh.2024.1378349

**Published:** 2024-05-28

**Authors:** Arto J. Hautala, Babooshka Shavazipour, Bekir Afsar, Mikko P. Tulppo, Kaisa Miettinen

**Affiliations:** ^1^Faculty of Sports and Health Sciences, University of Jyväskylä, Jyväskylä, Finland; ^2^Faculty of Information Technology, University of Jyväskylä, Jyväskylä, Finland; ^3^Research Unit of Biomedicine and Internal Medicine, Medical Research Center Oulu, Oulu University Hospital, University of Oulu, Oulu, Finland

**Keywords:** exercise, cardiac rehabilitation, coronary artery disease, coronary heart disease, artificial intelligence, health care costs, economic evaluation

## Abstract

**Introduction:**

Exercise-based cardiac rehabilitation (ECR) has proven to be effective and cost-effective dominant treatment option in health care. However, the contribution of well-known risk factors for prognosis of coronary artery disease (CAD) to predict health care costs is not well recognized. Since machine learning (ML) applications are rapidly giving new opportunities to assist health care professionals’ work, we used selected ML tools to assess the predictive value of defined risk factors for health care costs during 12-month ECR in patients with CAD.

**Methods:**

The data for analysis was available from a total of 71 patients referred to Oulu University Hospital, Finland, due to an acute coronary syndrome (ACS) event (75% men, age 61 ± 12 years, BMI 27 ± 4 kg/m2, ejection fraction 62 ± 8, 89% have beta-blocker medication). Risk factors were assessed at the hospital immediately after the cardiac event, and health care costs for all reasons were collected from patient registers over a year. ECR was programmed in accordance with international guidelines. Risk analysis algorithms (cross-decomposition algorithms) were employed to rank risk factors based on variances in their effects. Regression analysis was used to determine the accounting value of risk factors by entering first the risk factor with the highest degree of explanation into the model. After that, the next most potent risk factor explaining costs was added to the model one by one (13 forecast models in total).

**Results:**

The ECR group used health care services during the year at an average of 1,624 ± 2,139€ per patient. Diabetes exhibited the strongest correlation with health care expenses (*r* = 0.406), accounting for 16% of the total costs (*p* < 0.001). When the next two ranked markers (body mass index; *r* = 0.171 and systolic blood pressure; *r* = − 0.162, respectively) were added to the model, the predictive value was 18% for the costs (*p* = 0.004). The depression scale had the weakest independent explanation rate of all 13 risk factors (explanation value 0.1%, *r* = 0.029, *p* = 0.811).

**Discussion:**

Presence of diabetes is the primary reason forecasting health care costs in 12-month ECR intervention among ACS patients. The ML tools may help decision-making when planning the optimal allocation of health care resources.

## 1 Introduction

The impact of cardiovascular diseases extends far beyond mere statistics, directly correlating with elevated rates of mortality, morbidity, and frailty among those affected. These consequences, in turn, contribute significantly to the overall health care costs (1). Therefore, health care providers worldwide are required to target resources within the accessible and effective health services for the management of cardiovascular diseases especially coronary artery disease (CAD) (2).

Exercise-based cardiac rehabilitation (ECR) is recognized as a key component of comprehensive CAD management in international guidelines (3, 4). For example, the recent meta-analysis with 85 randomized controlled trials of 23,430 CAD patients showed that ECR reduced the risk of cardiovascular mortality, recurrent cardiac events, and hospitalizations, and improved health-related quality of life (5). Importantly, there is evidence showing that ECR is a dominant treatment option in comparison to usual care only in economic data analysis approaches (4, 5). Demonstrating the cost-effectiveness of ECR in operational planning and decision-making can assist decision-makers in allocating resources to treatments that offer patients the highest attainable health benefits while maintaining reasonable costs.

The implementation of machine learning (ML) techniques in clinical practice holds significant promise for optimizing health care resource utilization, ensuring both effectiveness and cost-efficiency (6). Recently, we utilized specific feature importance analysis to assess the predictive power of treated causal and modifiable risk factors at baseline in forecasting health care costs over a 12-month follow-up period for patients recovering from acute coronary syndrome (ACS) under usual care within the Finnish health care system. Our findings highlighted that a higher depression score emerged as the leading predictor of health care costs, succeeded by elevated levels of low-density lipoprotein (LDL) cholesterol and a reduced left ventricular ejection fraction (7).

Since the applicability of chosen ML tools was tested in the previous study and the ECR has shown marked improvements in risk factors among ACS patients, we tested if the primary predictive risk variables will be different when ACS patients in addition to usual care participate to the ECR intervention. We hypothesized that ECR in addition of usual care will modify the order of risk marker models compared to the usual care only. The specific aim of present study is to utilize ML tools to evaluate the predictive significance of specific risk factors for health care costs in patients with ACS who, in addition to receiving usual care, participated in a 12-month ECR program.

## 2 Methods

### 2.1 Study population

This research is a component of the EFEX-CARE (Effectiveness of Exercise Cardiac Rehabilitation) study, which has been officially registered with the Identifier Record NCT01916525 on ClinicalTrials.gov. The participants in the EFEX-CARE study were drawn from a consecutive series of ACS patients in the Division of Cardiology at the Oulu University Hospital. All the measurements were performed at the Oulu University Hospital. Each participant underwent coronary angiography to confirm the presence of CAD. A comprehensive description of the EFEX-CARE study population has been previously provided (8); however, to summarize, individuals with NYHA class ≥III, those scheduled for or undergoing emergency by-pass surgery, individuals with unstable angina pectoris, severe peripheral atherosclerosis, diabetic retinopathy or neuropathy, or those unable to engage in independent daily physical activities due to musculoskeletal issues were excluded from the study.

In the present study, we will show the health care costs for a 12-month follow-up and baseline risk marker data measured approximately two to 3 weeks after their hospital discharge for patients receiving usual care and participating to international guideline prescribed ECR. Originally the EFEX-CARE study was a randomized controlled trial, where the ACS patients were randomized to the usual care or ECR groups. In the original EFEX-CARE study 109 participants from the exercise training group were involved in the study. Finally, 78 individuals completed the study as planned. Drop-outs from the study were due to a lack of motivation, loss of interest, logistic problems, loss of time mainly because of work duties or health-related problems. Since data from all measured risk markers were needed for the present analysis, data for a total of 71 ECR patients was available. The research adhered to the CONSORT guidelines and was conducted in accordance with the Declaration of Helsinki. The local committee of research ethics for the Northern Ostrobothnia Hospital District approved the study protocol, and all participants provided written informed consent.

### 2.2 Assessment of patient characteristics, risk markers and health care costs

We gathered a range of health-related data through different methods. Briefly, body weight and height were measured to assess body composition. Blood pressure was measured in a supine position after a 10-min resting period, following current guidelines. Self-rated depression was assessed using the Depression Scale (DEPS) questionnaire (9). Data on smoking status, alcohol use disorders identification (AUDIT-C) (10), medication, history of acute myocardial infarction, and revascularization were obtained from hospital records and standard questionnaires. Left ventricular systolic function was assessed using 2-D echocardiography (Vivid 7, GE Health care, Wauwatosa, WI, United States). Blood samples were collected after a 12-h overnight fast to analyze plasma glucose, glycated hemoglobin (HbA1c), blood lipids, insulin, and high-sensitivity C-reactive protein using consistent methods at the Oulu University Hospital, Finland. An incremental symptom-limited maximal exercise test was conducted at the Oulu University Hospital using a bicycle ergometer (Monark Ergomedic 839 E, Monark Exercise AB, Vansbro, Sweden) to assess maximal physical exercise capacity (measured in metabolic equivalents: METs). Health-related quality of life was assessed using the 15D questionnaire (11) and completed by patients at the hospital before discharge.

For estimating health care costs, we considered specialized and primary health care services, as well as occupational health care services. Social security ID numbers were used to determine ambulatory care visits, treatment days, and external service usage, with costs calculated based on the Diagnosis Related Groups (DRG) classification. Data for primary health care services, including doctor visits, examinations, and in-ward treatment days, were obtained from electronic health registries using unique social security ID numbers. Information on home care and institutional care (e.g., assisted care homes) was gathered from registries. Additionally, we utilized the report of the Social Insurance Institute of Finland (KELA) (12) to estimate occupational health care service costs. All costs were considered in 2015 values, and no discounting was applied due to the one-year time horizon of the analysis.

### 2.3 Exercise-based cardiac rehabilitation

The ECR program started as soon as possible after hospital discharge, as suggested earlier (13). The 12-month ECR was planned according to the guidelines (14, 15) consisting of aerobic (30–40 min) and strength exercises (30–40 min) 3 to 5 times per week. The ECR group were invited to the Verve Rehabilitation Center in Oulu, Finland to begin a 12-month ECR program. During the initial 6 months, they attended the Cardiac Rehab gym equipped with aerobic and strength exercise devices (Smart Card system, Ab HUR Oy, Kokkola, Finland), once a week, where they received individual guidance from a physical therapist on both gym and the other exercises performed home-based. After 6 months, home-based ECR continued and only checkpoint visits to monitor the progression of exercise training were scheduled at 9 and 12 months. The intensity of the exercises was stated using the perceived ratings of exertion (RPE) scale from 6 to 20 (16) and targeted to the level between 12 and 15 RPE. Realized training load was calculated from the diaries (RPE x duration of each exercise session) (17). We refer interested readers to Hautala et al. (8) for a detailed intervention description.

### 2.4 Definition of predictive models

In predictive modeling, feature importance scores indicate the significance of input features in predicting the target variable (18). These scores guide feature selection, helping reduce computational costs and potentially enhance model performance. They offer valuable insights for improving predictive models through feature selection (19, 20) and dimensionality reduction (21, 22). Various methods exist, such as those based on statistical correlations and variances, but the choice of method should align with the specific variables and data types, necessitating the evaluation of multiple techniques for suitability. Based on our previous study (7), we performed the feature importance analysis including Cross Decomposition (23), Partial Least Squares Canonical Analysis (PLSC), Partial Least Squares based on Singular Value Decomposition (PLSSVD), Partial Least Square Regression (PLSRegression), Canonical Correlation Analysis (CCA), and Analysis of Variance (ANOVA) test relying on *p*-values for ranking.

Following the ranking of health care cost risk factors, we conducted a linear regression analysis to predict costs. This involved starting with the top-ranking risk marker and gradually adding the following best markers, resulting in the creation of 13 predictive models. Descriptive statistical analyses, including means, standard deviations (SDs), and proportions where relevant, were performed. We utilized the SPSS software (version 28, SPSS Inc., Chicago, IL, United States) for these predictive data analyses. Statistical significance was established with a *p*-value threshold of <0.05 for all tests.

## 3 Results

Table 1 displays the fundamental demographic features, clinical attributes, and medication usage patterns of the individuals included in the study. Interestingly, the monthly realized training volume, averaging at 15870 ± 7,758, surpassed the prescribed training volume of 10,740 ± 507 by a significant 51% (*p* < 0.0001). Furthermore, the average total cost per ACS patient over a 12-month follow-up period stood at 1624 ± 2,139 € for all causes.

**Table 1:** Baseline characteristics, health care costs and medication use (*n* = 71).

Variable	Exercise-based cardiac rehabilitation
Men, *n*	53 (75%)
Patients with T2D, *n*	15 (21%)
Age, year	61 ± 12
Weight, kg	82 ± 15
BMI, kg/m^2^	27.1 ± 4.2
Systolic BP, mmHg	136 ± 21
Diastolic BP, mmHg	76 ± 10
Exercise capacity, MET	6.1 ± 1.7
Quality of life, 15-D	0.92 ± 0.08
AUDIT-C for alcohol use	3.2 ± 2.4
Depression scale	4.8 ± 4.9
Current smokers, *n*	7 (10%)
Cost for all reasons, (€)	1,624 ± 2,139
History of AMI	
NSTEMI, *n*	33 (46%)
STEMI, *n*	29 (41%)
Revascularization	
PCI, *n*	61 (86%)
Earlier CABG, *n*	4 (6%)
Cardiac function	
LVEF, %	62 ± 8
CCS class	1.4 ± 0.6
Laboratory analyses	
HbA1c, %	5.9 ± 0.6
Fasting plasma glucose, mmol/l	5.9 ± 0.9
Total cholesterol, mmol/l	3.8 ± 0.8
HDL cholesterol, mmol/l	1.2 ± 0.3
LDL cholesterol, mmol/l	2.2 ± 0.7
Triglycerides, mmol/l	1.5 ± 1.3
hs-CRP, mg/l	1.8 ± 3.3
Medication	
Beta blockers, *n*	63 (89%)
ACEI or ARB, *n*	62 (87%)
Lipids, *n*	70 (99%)
Anticoagulants, *n*	71 (100%)
Calcium antagonists, *n*	12 (17%)
Nitrates, *n*	16 (23%)
Diuretics, *n*	11 (15%)

Values are means ± SD or the number of subjects (proportion); T2D, type 2 diabetes; BMI, body mass index; BP, blood pressure; MET, metabolic equivalent; 15-D, health-related quality of life; AUDIT-C, identifies at-risk drinkers for alcohol use; AMI, acute myocardial infarction; NSTEMI, non-ST segment elevation myocardial infarction; STEMI, ST segment elevation myocardial infarction; PCI, percutaneous coronary intervention; CABG, coronary artery by-pass grafting; LVEF, left ventricular ejection fraction; CCS, Canadian Cardiovascular Society grading of angina pectoris; HbA1c, glycated hemoglobin; HDL, high-density lipoprotein; LDL, low-density lipoprotein; hs-CRP, high-sensitivity C-reactive protein; ACEI, angiotensin conversion enzymes inhibitor; ARB angiotensin receptor blocker.

**Figure 1:**
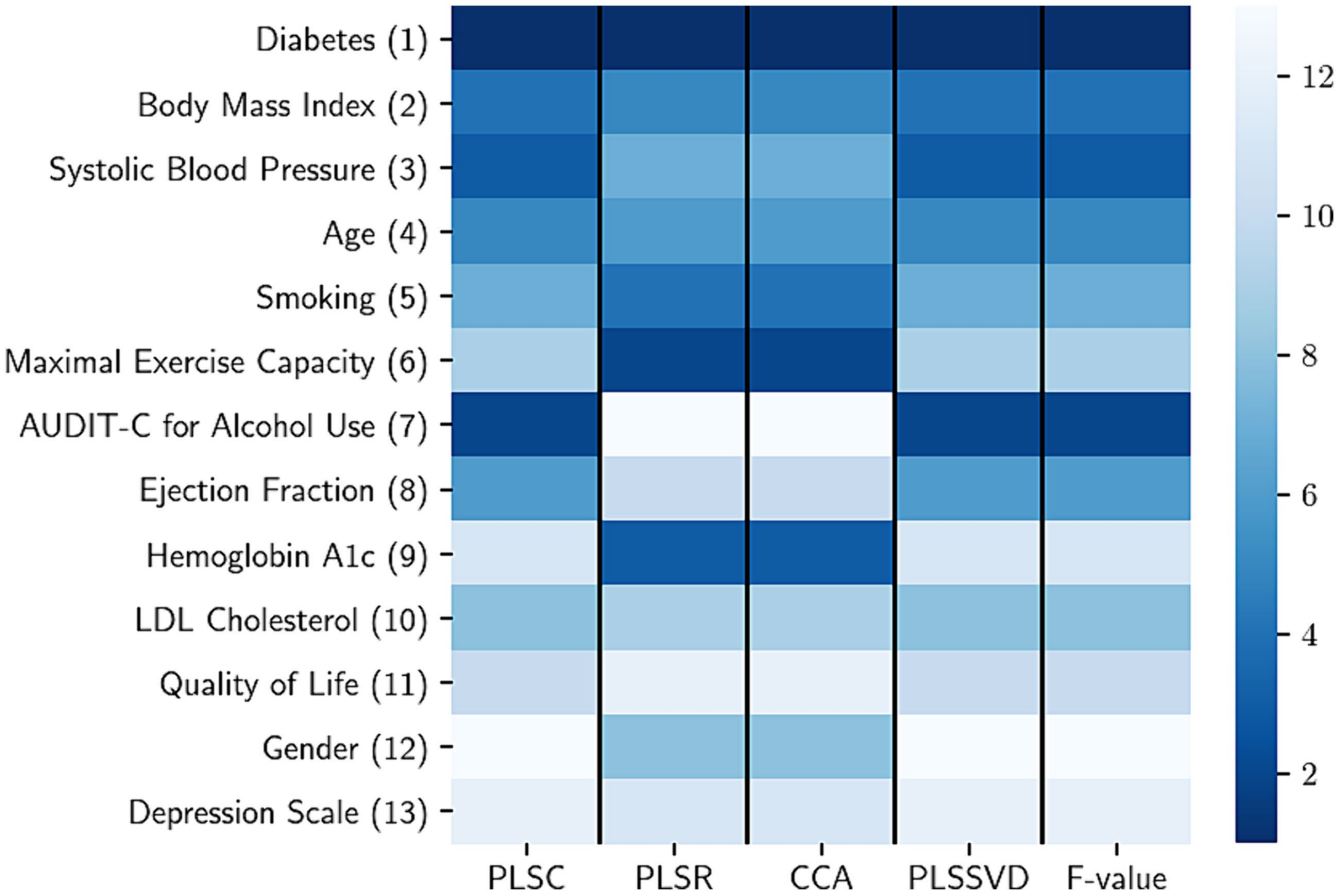
The rank aggregation process involves consolidating risk factors calculated using various methods, with each column representing a different feature selection method. In the heatmap, a lower rank value (indicated by a darker color) signifies a higher level of importance for the respective risk factor. The risk factors considered include LDL (low-density lipoprotein), PLSC (partial least squares canonical analysis), PLSR (partial least square regression), CCA (canonical correlation analysis), PLSSVD (partial least squares based on singular value decomposition), and *F* value obtained from the analysis of variance.

Figure 1 illustrates the hierarchy of risk factors utilized for forecasting health care costs. On the right side, a color scheme indicates the ranking (1–13) of these risk factors within each feature selection technique. In this heat map, a lower rank value is associated with a darker color, signifying the increased significance of the respective risk factor. The numbers enclosed in parentheses (1–13) present the overall ranking of each risk factor, considering its positions across multiple selection methods.

Table 2 provides an overview of the predictive models and their respective contributions to health care costs. It also illustrates the direction of each independent risk factor’s impact (positive or negative) through correlation values. Notably, diabetes emerged as the most influential predictor with a value of *r* = 0.406, attributing to 16% of the costs (*p* < 0.001). Upon incorporating the subsequent two highest-ranked markers, namely body mass index (*r* = 0.171) and systolic blood pressure (*r* = −0.162), the predictive capacity of the model increased to 18% for the costs (*p* = 0.004). On the other hand, the depression scale exhibited the lowest independent explanatory power among all 13 risk factors, contributing 0.1% (explanation value, *r* = 0.029, *p* = 0.811) to the overall model.

**Table 2:** Linear regression analysis models according to risk markers for prognosis of coronary artery disease at baseline for prediction of health care costs in exercise-based cardiac rehabilitation during one-year intervention.

Risk markers	R correlation	Model	R square	*p*-value
Diabetes	0.406	1	0.164	<0.001
Body mass index	0.171	2	0.166	0.002
Systolic blood pressure	−0.162	3	0.182	0.004
Age	0.144	4	0.185	0.008
Smoking	−0.138	5	0.188	0.016
Maximal exercise capacity	−0.094	6	0.189	0.032
AUDIT-C for alcohol use	0.085	7	0.221	0.022
Ejection fraction	0.061	8	0.240	0.023
HbA1c	0.070	9	0.283	0.011
LDL cholesterol	−0.054	10	0.283	0.019
Quality of life, 15-D	0.045	11	0.300	0.020
Gender	−0.039	12	0.300	0.033
Depression scale	0.029	13	0.307	0.044

The models were defined according to ranking analysis for well addressed causal and modifiable risk markers for prognosis of coronary artery disease at baseline. The Model 1 includes top one ranking risk marker Diabetes. The models from 2 to 13 are defined by entering the top second parameter (Body mass index) to the model. Accordingly, the next risk markers were added one-by-one to the defined models (3. Systolic blood pressure, 4. Age, 5. Smoking, 6. Maximal exercise capacity, 7. AUDIT-C for alcohol use, 8. Ejection fraction, 9. HbA1c, 10. LDL cholesterol, 11. Quality of life, 12. Gender and 13. Depression Scale).

## 4 Discussion

The results shown in the present study provide insights into the well-known risk factors collected from health care registries that influence health care costs predicted by ML algorithms, specifically in the context of patients with ACS in a 12-month ECR intervention. We found that diabetes is the primary contributing factor to health care costs followed by a higher body mass index. These findings highlight the significance of various demographic and clinical attributes in predicting health care expenditures, which can have important implications for health care providers, policymakers, and researchers.

### 4.1 Diabetes forecasting health care costs

Diabetes is one of the fastest-growing global health emergencies of the 21st century. The combination of diabetes with ACS enhances the risk for cardiovascular events emphasizing an interdisciplinary approach for a personalized treatment strategy including exercise training (24) to reduce each patient’s disease burden (25). Diabetes is one of the most influential predictors of health care costs associated with a myriad of complications, including cardiovascular issues, neuropathy, and kidney disease, all of which can substantially increase health care costs. For example, the total estimated cost of diagnosed diabetes in the U.S. in 2022 was 414$ billion, including 307$ billion in direct medical costs and 106$ billion in indirect costs attributable to diabetes (26). We found diabetes as the primary contributing risk factor for the health care costs, followed by body mass index and systolic blood pressure with our ACS patients, although they obeyed carefully exercise training prescriptions and exceeded their prescribed training volume by a significant 51%. It is also important to mention that blood glucose level in terms glycated hemoglobin (HbA1c) and fasting plasma glucose were well balanced according to treatment targets in our patients. Nevertheless, diabetes itself showed to be the main contributor for the health care costs.

### 4.2 Exercise intervention changes the risk marker profile for health care costs

In our previous study we tested the applicability of ML tools to predict health care costs in a group of ACS patients treated with usual care only. The chosen methods for feature importance demonstrated their effectiveness in ranking established risk markers, determining the most critical primary targets among them. This aids in identifying key contributors to healthcare costs. We observed that depression, expressed as the higher DEPS score, was the primary contributing factor of health care costs in a 12-month follow-up (7). Interestingly, for the ECR group in the present study, the DEPS score had the lowest explanatory power in predicting health care costs without practically no contribution (0.1%). This is interesting an finding, since not only in terms of health care costs, but understanding that psychosocial risk factors, such as depression, have shown their significance in affecting cardiovascular prognosis, treatment adherence, quality of life, and even sudden cardiac death (27, 28). We also confirmed that the ACS patients involved in usual care did not differ from the ECR group in the present study at baseline in any clinical variables such as diabetes, DEPS score, or any other risk factor, or medications (Supplementary Table S1). Furthermore, it is notable that all ACS patients in the EFEX-CARE study were willing to participate and were at baseline randomized into the ECR and usual care groups.

**Figure 2:**
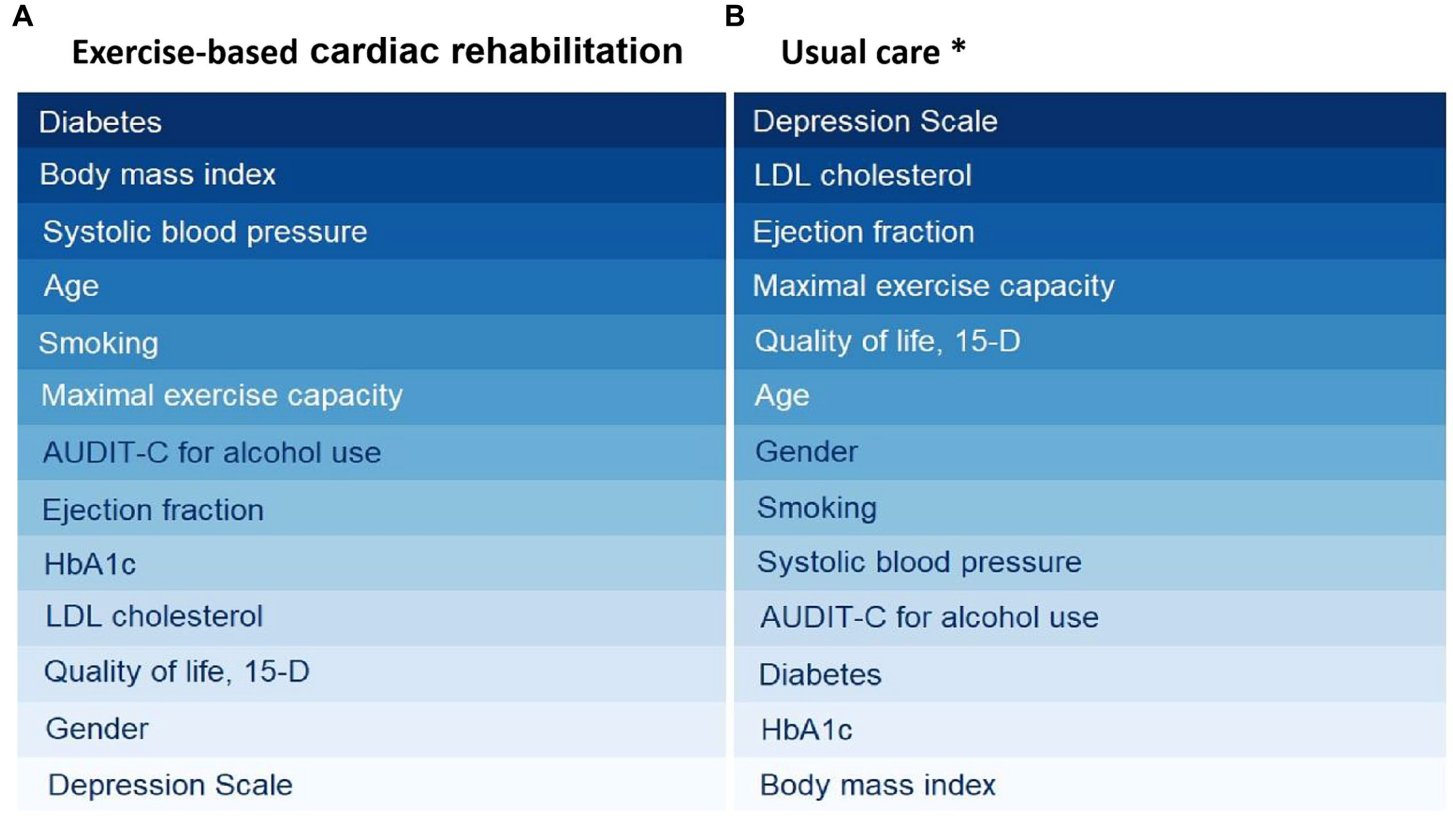
Risk analysis algorithms (cross-decomposition algorithms) to rank risk factors in exercise-based cardiac rehabilitation **(A)** and usual care **(B)**. A darker color signifies a higher level of importance for the respective risk factor. ^*^Usual care group ranking has been published earlier by Hautala et al. (7).

In addition to medical treatment, exercise is a key component of comprehensive CAD management (5, 14). It is also well-documented that exercise is efficacious in treating depression and depressive symptoms. Therefore, it should be offered as an evidence-based treatment option to individuals with a diagnosis of a major depressive disorder or those with depressive symptoms (29) and similarly with cardiovascular disease patients suffering from mental health disorders (30). It is important to note that the DEPS scale we used in this study is a self-report scale that assesses the severity of depressive symptoms, and it should not be used as the sole basis for diagnosing depression (31). However, it seems that the ECR changes markedly the important risk marker profile measured at baseline compared to the group of patients treated in usual care only when predicting health-care costs in a 12-month time course (Figure 2).

In the present study, we used the selected feature importance methods to find the most preferred first-order targets of risk markers to contribute to health care costs. We also assessed if the order of the leading predictive risk marker will change if we remove one-by-one the risk markers ranked from 6 to 13. Despite excluding the risk markers from the feature importance analysis, the order of the five leading markers remained the same. Therefore, in addition to selected feature importance analysis tools, the selected risk markers included were relevant and valid to the performed analysis.

### 4.3 Strengths and limitations

We believe that the strength of this study lies in its utilization of hospital records to determine the use of health care services, as opposed to relying on patient self-reports, thus minimizing recall bias. Furthermore, the study conducted a comprehensive assessment of baseline patient characteristics, encompassing clinical status, medication usage, thorough laboratory analysis and realized exercise training analysis from diaries. However, it is important to acknowledge a limitation in the study, namely the small and possibly selectively chosen patient sample in the EFEX-CARE study, which may restrict the generalizability to a broader population of ACS patients with significant co-morbidities. Nevertheless every participant in the EFEX-CARE study expressed a willingness to take part and was subsequently randomized into either the ECR or usual care groups.

Our findings demonstrated that when all 13 markers were combined, this model could only predict 31% of the costs. While this may initially appear low, it is worth noting that the proprietary nature of economic data and the diverse sources of health care cost components may partly account for these results. For instance, we were able to analyze direct health care costs but not indirect costs stemming from reduced work productivity due to health issues. An open question remains: can the predictive value be enhanced by incorporating additional variables? This question presents a potential avenue for future research. Additionally, although we assessed various feature importance methods to establish their stability, the relatively small sample size and multicollinearity among risk factors raise some caution in interpreting and generalizing the results. However, we believe that the proposed methodology holds promise for application in medical and health care settings where patient risk profiles and health care costs over a specific period need to be evaluated.

## 5 Conclusion

The study’s findings contribute to the growing body of knowledge on risk factors influencing health care costs in ACS patients who participated in guideline-prescribed exercise training intervention. The results highlight the importance of patient engagement, the impact of diabetes, and the significance of modifiable risk factors like BMI and blood pressure in health care costs.

This information can guide risk assessment in clinical practice and help identify high-risk patients who may benefit from targeted interventions. By understanding the relative importance of different risk factors, health care providers can tailor their care plans to address specific patient needs, potentially reducing health care costs while improving patient outcomes. These insights can inform health care policies, clinical guidelines, and interventions aimed at improving patient care and reducing the economic burden of ACS. However, further research and validation of these findings in diverse patient populations are needed to enhance their generalizability and utility in clinical practice.

## Data availability statement

The original contributions presented in the study are included in the article/Supplementary material, further inquiries can be directed to the corresponding author.

## Ethics statement

The studies involving humans were approved by the local committee of research ethics for the Northern Ostrobothnia Hospital District, Oulu, Finland. The studies were conducted in accordance with the local legislation and institutional requirements. The participants provided their written informed consent to participate in this study.

## Author contributions

AH: Conceptualization, Data curation, Formal analysis, Funding acquisition, Investigation, Methodology, Project administration, Resources, Supervision, Validation, Visualization, Writing – original draft, Writing – review & editing. BS: Conceptualization, Data curation, Formal analysis, Investigation, Methodology, Project administration, Software, Validation, Visualization, Writing – original draft, Writing – review & editing. BA: Conceptualization, Data curation, Formal analysis, Investigation, Methodology, Project administration, Software, Validation, Visualization, Writing – original draft, Writing – review & editing. MT: Conceptualization, Data curation, Formal analysis, Investigation, Methodology, Project administration, Supervision, Validation, Visualization, Writing – original draft, Writing – review & editing. KM: Conceptualization, Data curation, Formal analysis, Investigation, Methodology, Project administration, Software, Supervision, Validation, Visualization, Writing – original draft, Writing – review & editing.
