# Supplementary material for: Machine learning models for assessing risk factors affecting health care costs: 12-month exercise-based cardiac rehabilitation
Source: Front Public Health. 2024 May 28;12:1378349. doi: 10.3389/fpubh.2024.1378349 (PMC11165052; doi:10.3389/fpubh.2024.1378349)
Supplement: Supplementary file 1 [file Table_1.DOCX]

Supplement Table. Comparison of the baseline characteristics, health care costs and medication use between exercise-based cardiac rehabilitation and usual care groups. *Usual care group characteristics has been published earlier by Hautala et al. (2023).

| **Variable** | **Rehabilitation n=71** | **Usual care***  **n=65** | **p-value** |
| --- | --- | --- | --- |
| Men, n | 53 (75%) | 46 (71%) | 0.701 |
| Patients with T2D, n | 15 (21%) | 11 (17%) | 0.663 |
| Age, year | 61 ± 12 | 65 ± 9 | 0.599 |
| Weight, kg | 82 ± 15 | 83 ± 14 | 0.709 |
| BMI, kg/m^2^ | 27.1 ± 4.2 | 28.0 ± 4.3 | 0.910 |
| Systolic BP, mmHg | 136 ± 21 | 137 ± 22 | 0.745 |
| Diastolic BP, mmHg | 76 ± 10 | 78 ± 11 | 0.245 |
| Exercise capacity, MET | 6.1 ± 1.7 | 5.6 ± 1.7 | 0.073 |
| Quality of life, 15-D | 0.92 ± 0.08 | 0.90 ± 0.08 | 0.254 |
| AUDIT-C for alcohol use | 3.2 ± 2.4 | 2.9 ± 2.4 | 0.487 |
| Depression Scale | 4.8 ± 4.9 | 4.6 ± 5.3 | 0.853 |
| Current smokers, n | 7 (10 %) | 8 (12 %) | 0.786 |
| **Total average health care cost per patient** | | | |
| Cost for all reasons, (€) | 1624 ± 2139 | 2601 ± 5378 | 0.159 |
| **History of AMI** |  |  |  |
| NSTEMI, n | 33 (46%) | 45 (51%) | 0.679 |
| STEMI, n | 29 (41%) | 22 (34%) | 0.258 |
| **Revascularization** |  |  |  |
| PCI, n | 61 (86%) | 55 (85%) | 0.999 |
| earlier CABG, n | 4 (6%) | 8 (12%) | 0.229 |
| **Cardiac function** |  |  |  |
| LVEF, % | 62 ± 8 | 62 ± 7 | 0.922 |
| CCS class | 1.4 ± 0.6 | 1.6 ± 0.6 | 0.117 |
| **Laboratory analyses** |  |  |  |
| HbA1c, % | 5.9 ± 0.6 | 6.0 ± 0.8 | 0.275 |
| Fasting plasma glucose, mmol/l | 5.9 ± 0.9 | 6.0 ± 1.0 | 0.553 |
| Total cholesterol, mmol/l | 3.8 ± 0.8 | 3.8 ± 0.7 | 0.762 |
| HDL cholesterol, mmol/l | 1.2 ± 0.3 | 1.2 ± 0.3 | 0.507 |
| LDL cholesterol, mmol/l | 2.2 ± 0.7 | 2.1 ± 0.7 | 0.759 |
| Triglycerides, mmol/l | 1.5 ± 1.3 | 1.3 ± 0.6 | 0.264 |
| hs-CRP, mg/l | 1.8 ± 3.3 | 2.7 ± 6.0 | 0.292 |
| **Medication** |  |  |  |
| Beta blockers, n | 63 (89%) | 56 (86%) | 0.796 |
| ACEI or ARB, n | 62 (87%) | 54 (83%) | 0.629 |
| Lipids, n | 70 (99%) | 64 (98%) | 0.999 |
| Anticoagulants, n | 71 (100%) | 64 (98%) | 0.478 |
| Calcium antagonists, n | 12 (17%) | 17 (26%) | 0.213 |
| Nitrates, n | 16 (23%) | 18 (28%) | 0.554 |
| Diuretics, n | 11 (15%) | 15 (23%) | 0.283 |

Values are means ± SD or the number of subjects (proportion); T2D, type 2 diabetes; BMI, body mass index; BP, blood pressure; MET, metabolic equivalent; 15-D, health-related quality of life; AUDIT-C, identifies at-risk drinkers for alcohol use; AMI, acute myocardial infarction; NSTEMI, non-ST segment elevation myocardial infarction; STEMI, ST segment elevation myocardial infarction; PCI, percutaneous coronary intervention; CABG, coronary artery by-pass grafting; LVEF, left ventricular ejection fraction; CCS, Canadian Cardiovascular Society grading of angina pectoris; HbA1c, glycated hemoglobin; HDL, high-density lipoprotein; LDL, low-density lipoprotein; hs-CRP, high-sensitivity C-reactive protein; ACEI, angiotensin conversion enzymes inhibitor; ARB angiotensin receptor blocker.
